# Applying Convolutional Neural Networks to Predict the ICD-9 Codes of Medical Records

**DOI:** 10.3390/s20247116

**Published:** 2020-12-11

**Authors:** Jia-Lien Hsu, Teng-Jie Hsu, Chung-Ho Hsieh, Anandakumar Singaravelan

**Affiliations:** 1 Department of Computer Science and Information Engineering, Fu Jen Catholic University, New Taipei City 242062, Taiwan; gt810034@gmail.com; 2 Department of General Surgery, Shin Kong Wu Ho-Su Memorial Hospital, Taipei 111045, Taiwan; M012363@ms.skh.org.tw; 3 Graduate Institute of Applied Science and Engineering, Fu Jen Catholic University, New Taipei City 242062, Taiwan; 408068080@mail.fju.edu.tw

**Keywords:** diagnosis code prediction, convolutional neural network, ICD-9, medical record

## Abstract

The International Statistical Classification of Disease and Related Health Problems (ICD) is an international standard system for categorizing and reporting diseases, injuries, disorders, and health conditions. Most previously-proposed disease predicting systems need clinical information collected by the medical staff from the patients in hospitals. In this paper, we propose a deep learning algorithm to classify disease types and identify diagnostic codes by using only the subjective component of progress notes in medical records. In this study, we have a dataset, consisting of about one hundred and sixty-eight thousand medical records, from a medical center, collected during 2003 and 2017. First, we apply standard text processing procedures to parse the sentences and word embedding techniques for vector representations. Next, we build a convolution neural network model on the medical records to predict the ICD-9 code by using a subjective component of the progress note. The prediction performance is evaluated by ten-fold cross-validation and yields an accuracy of 0.409, recall of 0.409 and precision of 0.436. If we only consider the “chapter match” of ICD-9 code, our model achieves an accuracy of 0.580, recall of 0.580, and precision of 0.582. Since our diagnostic code prediction model is solely based on subjective components (mainly, patients’ self-report descriptions), the proposed approach could serve as a remote and self-diagnosis assistance tool, prior to seeking medical advice or going to the hospital. In addition, our work may be used as a primary evaluation tool for discomfort in the rural area where medical resources are restricted.

## 1. Introduction

In recent years, deep learning techniques have evolved and are used with good results in a wide variety of fields including speech recognition [1], computer vision [2], bioinformatics [3], and natural language processing [4,5]. In addition, the Convolutional Neural Networks are not only often used in computer vision but have also been successfully applied to sentence classification tasks with good results [6]. During medical diagnosis, the physician will assign a particular disease code (i.e., ICD-9) that corresponds to the patient’s condition. In this paper, we would like to train a neural network model using deep learning algorithms and data mining techniques to assist physicians in selecting the appropriate disease code. Since our diagnostic code prediction model is solely based on the subjective component of the progress note, the proposed approach could serve as a remote and self-diagnose assistance tool, by providing a description of their condition, prior to seeking medical advice.

### 1.1. Electronic Medical Record

An electronic medical record (EMR) is a multimedia file or a set of files that can be electronically stored, transmitted and managed, and may include texts, images, audios and videos that comprise the patient profile, nurse’s notes, progress notes, laboratory test results, allergies, past medical history, family history, etc. [7]. Harerimana et al. [8] listed the most feasible use cases of deep learning combined with electronic health record (EHR). Hospitals apply various customized EHRs that are enclosed of different kinds of unstructured elements to help overcome problems associated with traditional paper-based medical records, including difficulties in identification, inconsistent formatting, and inconvenient transmission. Thus it improves the quality of medical services and the efficiency of hospital operations and management. Yin et al. [9] state there has been a fast development in diversity of EHR during the last decades. This growth makes it feasible to apply prediction models to enhance the quality of clinical care.

The EMR content can be divided into outpatient records, emergency records and inpatient records. Inpatient medical records mainly include a red sheet, admission notes, inpatient progress notes, consultation notes, operation notes, anesthesia records, orders, and discharge summaries. The hospitalization history is a record of changes to the patient’s condition kept by medical staff following the patient’s admission to the hospital, with information updated by the resident on duty including treatment records, weekly summaries, on-service notes, and off-service notes. Changes to the patient’s condition are recorded in detail, including symptoms, physical examinations, treatment strategies, treatment responses, diagnosis and treatment plans. If the patient’s condition is unstable, medical staff may update the patient’s record multiple times each day. The recording process is described as follows.

**Figure 1:**
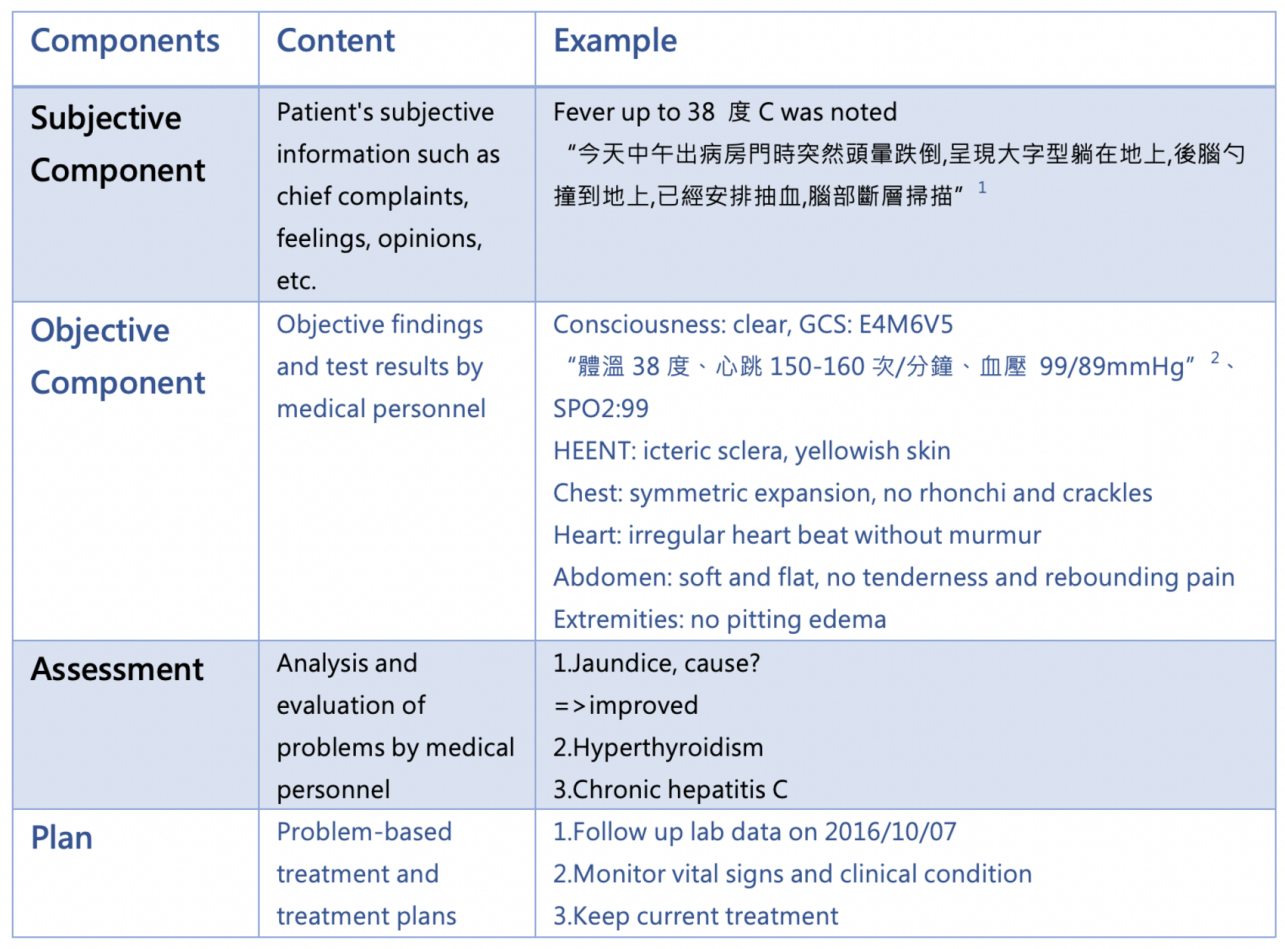
An example of progress note. 1. The (original) Subjective Component data was written in Chinese. The translation is “Fever up to 38 °C was noted. ‘I felt dizzy this afternoon as I walked out of the ward and fell onto the ground prostrate. I hit my hindbrain. The doctor has arranged a blood test and CT scan’.”. 2. The (original) Objective Component data was written in Chinese. The translation is “body temperature 38°; heart rate 150–160/min; blood pressure 99/89 mmHg.”.

The patient history (i.e., progress note) is kept using the Problem-Oriented Medical Record (POMR) method, focusing on Dysfunction which is also used as the record’s title. The medical record’s formatting is referred to as the SOAP format: subjective component (primarily the subjective data about the patient including patient complaints, feelings, and opinions), objective component (objective findings from tests administered by medical personnel), assessment- analysis and evaluation of the problem by medical staff), and plan (treatment plan for the problem). Figure 1 shows an example of a progress note.

### 1.2. International Statistical Classification of Diseases and Related Health Problems (ICD)

The ICD (International Statistical Classification of Diseases and Related Health Problems), maintained by the World Health Organization (WHO), is a system of categorization and coding for disease [7]. Generally, ICD codes are used to categorize the state of the disease during hospital occasions. Public health decisions are made based on the ICD codes which are used to illustrate the patient and expanding disease registries [10]. The ICD is updated every ten years, and the current version is ICD-10. Many countries use the ICD as the basis for collecting death and disease statistics. To respond to conditions in different countries and their medical conditions, many versions of the ICD have been drafted, such as the ICD-10-CM in the United States and the ICD-10-CA in Canada. In 1995, the government of Taiwan mandated the use of ICD-9-CM as the basis for medical institutions declaring health care medical expenses under Taiwan National Health Insurance (Taiwan NHI). In 2010, ICD-10-CM was adopted as new updates. In this paper, we use ICD-9 as the basis for model construction and prediction, since our dataset of the medical record is kept using ICD-9.

**Figure 2:**
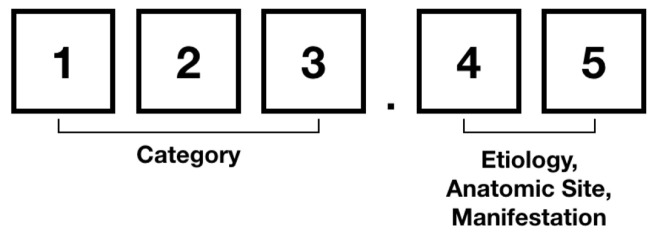
The structure of International Statistical Classification of Disease and Related Health Problems (ICD)-9 code.

**Figure 3:**
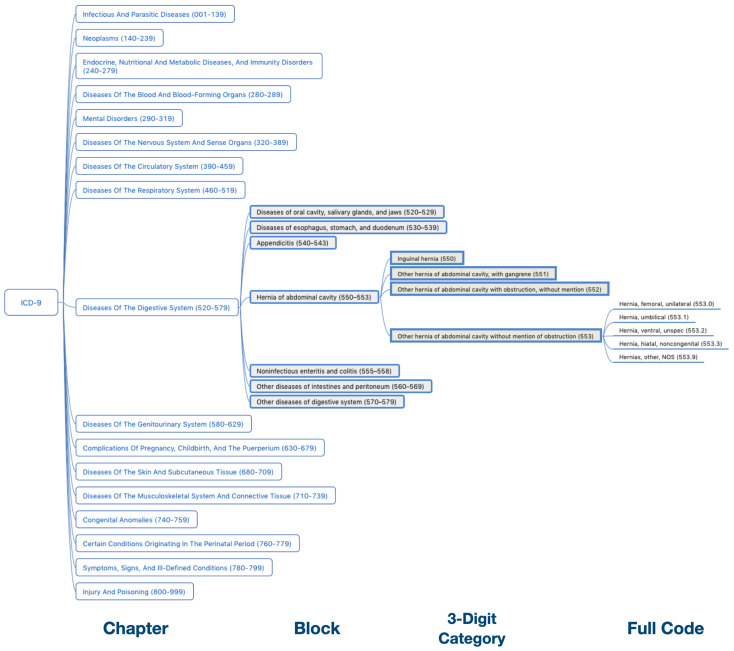
The ICD-9 hierarchical tree for Hernia.

The ICD-9 consists of three volumes: a Tabular List of diagnosis codes, an Alphabetic Index of diagnosis code, and Procedures Code for surgical, diagnostic, and therapeutic procedures. The ICD-9 codes embody about 10,000 different codes including 17 chapters of disease codes and two chapters for trauma and supplemental classifications (i.e., E and V codes). The Taiwan ICD-9 is adapted from the original ICD-9 used in the United States, in which the 17 chapters of the ICD-9 can be subdivided into 135 blocks, each consisting of an array of disease codes. Note that, the the core category is represented in the first three digits of ICD-9 code, and additional details are indicated at the fourth digit or the optional fifth digit. In reference to Figure 2, the first three digits (Three-Digit Category) indicate the disease category and are separated by a decimal point from the last two digits (i.e., subcategory) which indicate the cause, anatomic site, and the manifestation. Figure 3 illustrates the ICD-9 hierarchy tree for Hernia.

### 1.3. Deep Learning

Deep learning is a branch of machine learning and is one of the most promising areas in developing artificial intelligence. Deep learning algorithms derive from artificial neural networks that make use of multiple processing layers to extract the features of the original data and then classify these features using the neural network [5]. Many deep learning models have been proposed, including Deep Neural Networks, Convolutional Neural Networks, and Recurrent Neural Networks. The deep learning models have been widely applied to medical applications. For example, Gao et al. [11] integrate deep learning, word embedding and EHR data to predict Neonatal Encephalopathy before birth of a baby.

The convolutional neural network used in this paper is explained below. The convolutional neural network consists of Convolutional Layers, Pooling Layers, and Full-Connected Layers. Convolution is the main mechanism by which features are extracted in convolutional neural networks by exploiting the weight of the filter and the inner product of the input data. The filter with strides back and forth on the input data to generate a new feature map. The weight of the convolved filter and parameters of the fully-connected neurons are updated in the training process of the neural network using the backpropagation algorithm and the optimization algorithm. The pooling layer is usually connected to the convolutional layer. Its main function is to compress the data and capture features. Pooling is similar to convolution in that filter shows strides over the input data. The difference is in pooling filtering, where the device will select the maximum or average value from the input data. Common pooling algorithms include max pooling, k-max pooling and average pooling. Finally, following feature extraction and data compression, feature classification is performed by the fully-connected layer to obtain the prediction results.

Based on the fact that convolution filter can extract local features [12], the convolutional neural network could be applied to solve natural language processing problems [4,13]. Kim [6] designed a convolutional neural network to classify sentences by mapping words in the sentence through Word2Vec into a matrix as the input data (assuming *n* words in a sentence, the dimension of the word vector is *k*, and the size of the mapping matrix is *n*-by-*k*). Word embedding, also referred to as Word2Vec, is a technique of distributed word representation by mapping all words in a text to real-valued vectors in the dimensional space. This approach of unsupervised learning algorithm has been widely used in the field of natural language processing to obtain the similarity of words from a corpus. The weight of the word vector is divided into static and non-static, depending on whether it will be adjusted using the back-propagation algorithm during the convolutional neural network model training process. Previously, convolutional neural networks were used for image recognition using the RGB color channel. In one experiment (CNN-multichannel), Kim [6] used two channels to represent the same input data for static and non-static weights. After the data were input into the convolutional layer, the filter performed a one-dimensional convolution to generate a one-dimensional feature matrix. The feature matrix was then input into the Max Pooling Layer to extract the most important features, finally outputting the characteristics of all the pooled layers, which was then input into the fully-connected layer for classification. Kim added the Dropout mechanism to the fully-connected layer. The Dropout process multiplies each input data by a Bernoulli random variable during forward propagation. Accuracy can be improved and overfitting avoided by setting the connection weight to zero under a certain probability (the probability is set by a hyperparameter) [14].

### 1.4. Deep Learning for Disease Prediction

With the development of deep learning technologies, many researchers have applied hospital EMRs or medical databases for disease prediction by using deep learning algorithms to analyze highly complex data sets for assessing the risk and incidence of individual diseases. The systematic review [15] established the importance of electronic health records and also reported the cost efficiencies associated with electronic health records. The review clarifies investment in electronic health records which leads to developed health outcomes for the consumers and effective emergency departments. In the study by Gangavarapu et al. [16], the authors proposed a vector space and topic modeling-based approach applied to shape the raw clinical data by exploiting the data in the nursing notes. In addition, the authors use unstructured nursing notes to classify the ICD-9 group code. Gangavarapu et al. [17] also used clinical nursing notes to predict ICD code for long-term disease. Cheng et al. [18] exploited structured and unstructured hospital data, including basic patient profile (age, gender, and lifestyle), clinical examination data and physician diagnosis to predict the incidence of cerebral infarction, and achieved a high accuracy of 0.948.

Huang et al. [19] proposed a method to assign a ICD-9 code to track a patient’s medical history and also for billing purposes. The method followed clinical notes to predict the target ICD-9 code. Clinical notes consist of the medical history of patients, patient’s comments during the interaction, doctor’s observation note. From their study, the model identified the top-10 ICD-9 codes with the $F_{1}$ score of 0.6957 and accuracy of 0.8967. In addition, the proposed model achieved the top-10 ICD-9 categories with the $F_{1}$ score of 0.7233 and the accuracy of 0.8967. The process of assigning ICD-9 code may automatically trigger the clinical process effectively. In addition, the stages of semantic analysis have speeded up to help the clinician diagnose and develop the health care system. Patients may have more than one medical problems, which leads to multiple medical diagnoses. In the study by Samonte et al. [20], the proposed method of Enhanced Hierarchical Attention Network (EnHAN) followed topical word embedding and word embedding to solve this multi-class labeling and multi-label classification approach. This approach achieved a high accuracy of 0.841. Moons et al. [21] have applied multiple deep learning approaches on the classification of ICD-9 code. Their deep learning approaches utilized the discharge summary for the classification in which self-attention, regularization, and the loss function with attention included a convolutional neural network showed to be valuable. In the study of multi-label classification of ICD coding [22], the authors used Word2Vec to calculate the vector of the discharge summary. According to their study CNN achieved 76% by micro $F_{1}$ in label-to-chapter. Suo et al. [23] used convolutional neural networks to create a model to predict diabetes mellitus, obesity, and chronic obstructive pulmonary disease with accuracy up to 0.74. Cheng et al. [24] also used convolutional neural networks to establish a model to predict future recurrence of chronic heart failure and chronic obstructive pulmonary disease. In this study by Obeid et al. [25], the authors attempted to identify self-harm and suicide using clinical notes. The notes combined a variety of note types, including progress notes, plan of care notes, emergency department provider notes, history and physical notes, and consult notes. In reference to Table 1, we summarize the performance of work on ICD code prediction. It seems that the accuracy of our work is not as high as the previous work. However, in this study, we have the dataset containing 2017 distinct ICD-9 codes, which is more than those in previous work.

Note that the authors in [15,16,17,18,19,20,21,22,23,24,25] used patient profile, clinical examination reports and physician diagnosis results to establish models for the prediction of certain diseases that are commonly seen or with high mortality rates. On the contrary, in this paper, we only make use of patients’ self-report data (i.e., the subjective component in the progress note of EMR) to establish a predictive model for a variety of diseases. Prior to going to the hospitals, patients’ self-report data need not be taken from a physician’s diagnosis or professional clinical test, thus allowing patients to remotely self-diagnose a wide range of conditions based on the description of their symptoms. We can provide proper disease classification information to a patient by our text mining technique. When patients are admitted to hospital, our approach has the potential to inform physicians of unsuspected patient conditions while physicians are writing notes. Our work can also assist physicians in quickly make proper ICD coding. In the future, it is possible to apply our work on massive coding demand situations like outpatient department notes or insurance declaration.

## 2. Methods

**Figure 4:**
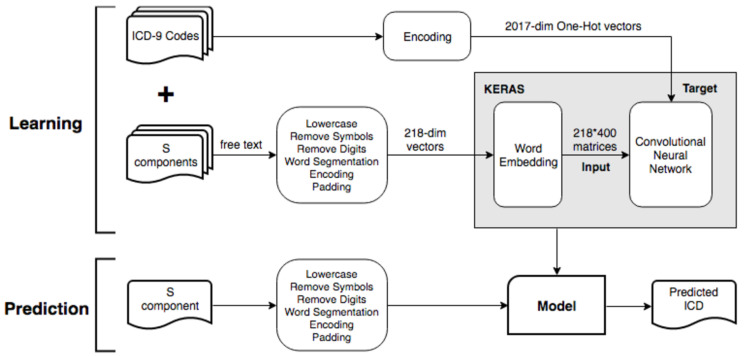
The system architecture.

In reference to Figure 4, we illustrate the system architecture for data cleaning, preprocessing and design pattern of neural network models for ICD-9 code prediction.

UTF8bsmi

### 2.1. The Data

**Figure 5:**
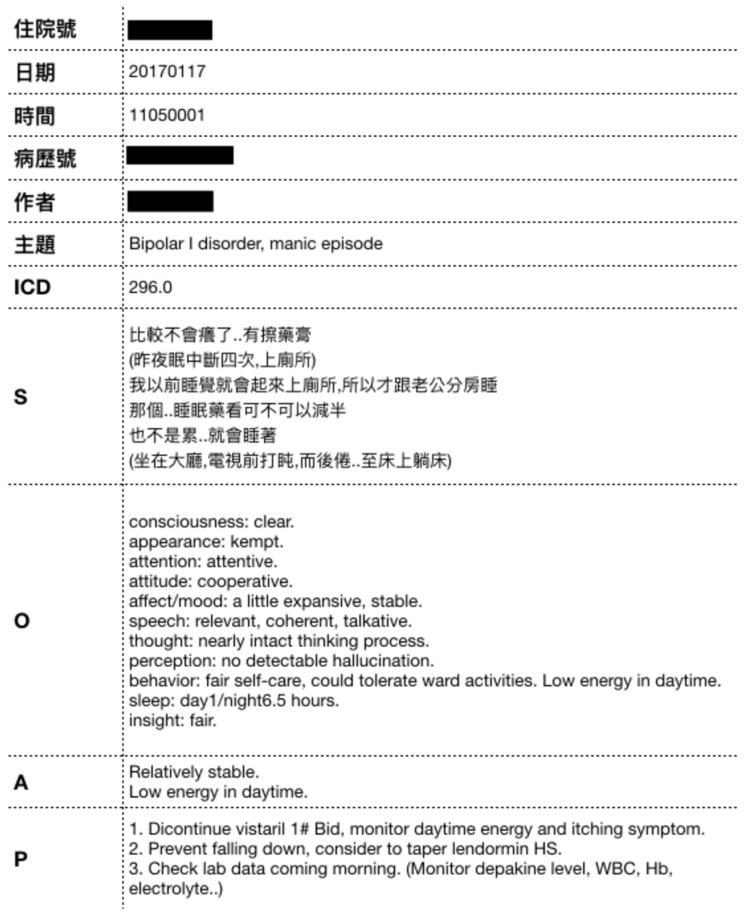
An example of medical record. The (original) Subjective Component in this example was written in Chinese. The translation is “It’s less itchy now… with the ointment. I woke up four times for bathroom last night. I have such a habit, so my husband and I sleep in separate rooms. It is okay to prescribe the drug to only half of the current volume. It’s not that I get tired easily, but I do doze off in the living room, before the TV, and I try myself to bed slowly”.

**Figure 6:**
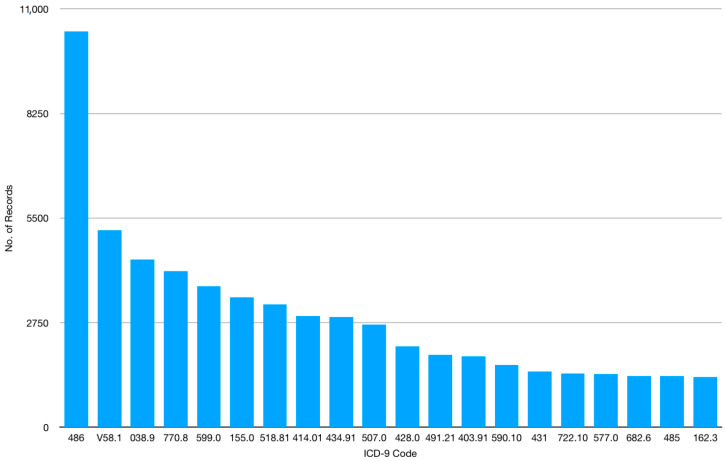
The distribution of the top 20 of most prevalent diseases with the highest record numbers.

**Figure 7:**
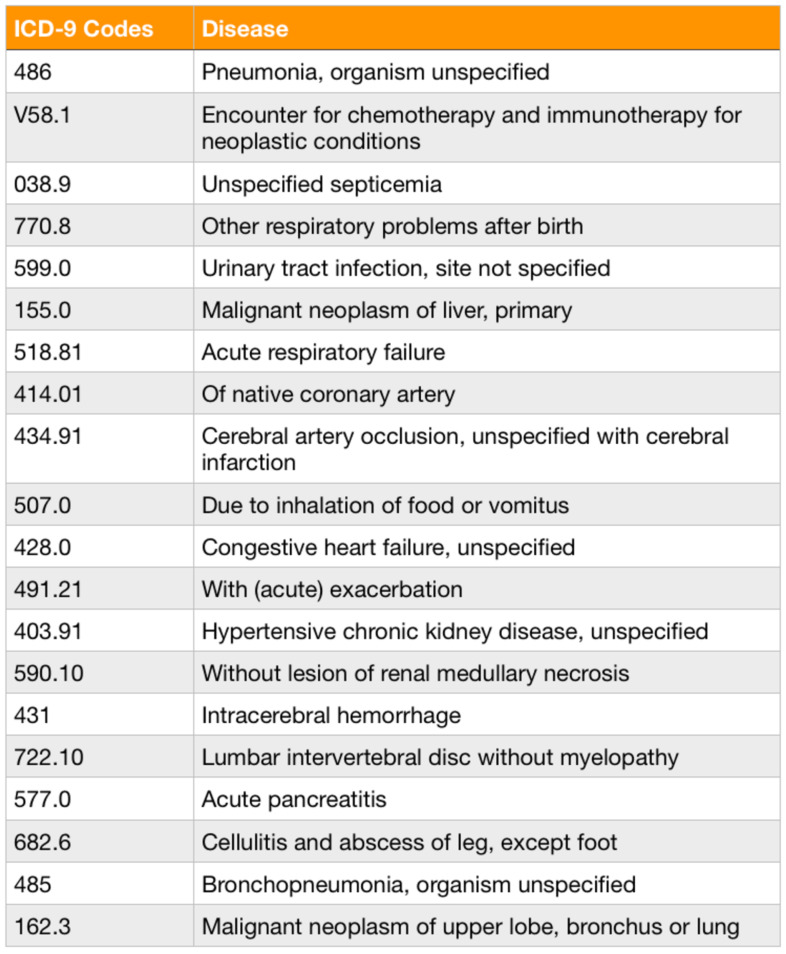
The top 20 of most prevalent diseases in our dataset.

The data used in this study are taken from EMR Progress Notes from a medical center from 2003 to 2017. The data set includes 168,186 medical records, each consisting of 11 fields: Inpatient ID, Date), Time, Medical Record ID, Author, Subject, ICD-9 code, Subjective Component, Objective Component, Assessment and Plan. In our dataset, there are 2017 different diseases in terms of distinct ICD-9 code. The subjective components are free text, which is self-reported by patients. Figure 5 shows an example of a medical record. Note that the original Subject Component in this example was written in Chinese. The translation is “It’s less itchy now … with the ointment. I woke up four times for bathroom last night. I have such a habit, so my husband and I sleep in separate rooms. It is okay to prescribe the drug to only half of the current volume. It’s not that I get tired easily, but I do doze off in the living room, before the TV, and I try myself to bed slowly”. Figure 6 and Figure 7 show the distribution and listing of the top 20 most prevalent diseases, respectively.

### 2.2. Preprocessing

The original medical records contain both Chinese and English text. To unify the English text, all English characters were rendered in lowercase, after we filtered out all half-width and specialized punctuation and numerals [26], leaving only Chinese text and lowercase English text for the following word segmentation by using the jieba package (https://github.com/fxsjy/jieba).

**Figure 8:**
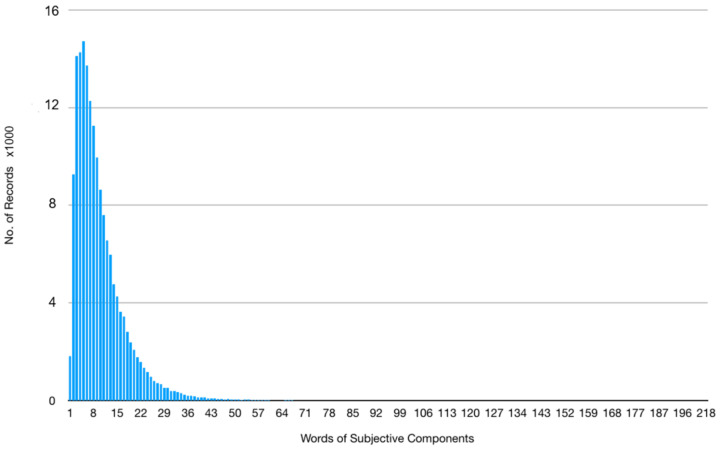
The distribution of word count in the subjective component.

After text cleaning and word segmentation, in our dataset, there are 30,236 distinct words. In reference to Figure 8, The length of the S component ranges from 1 to 218 words. To unify the length of the S component, we set the length of each S component to 218 words, with missing words in sentences shorter than 218 words padded by zero. As a result, each document (i.e., the S component) will be represented as a 218-dimensional document vector, associated with an ICD-9 code.

### 2.3. Problem Design and Experimental Setting

**Figure 9:**
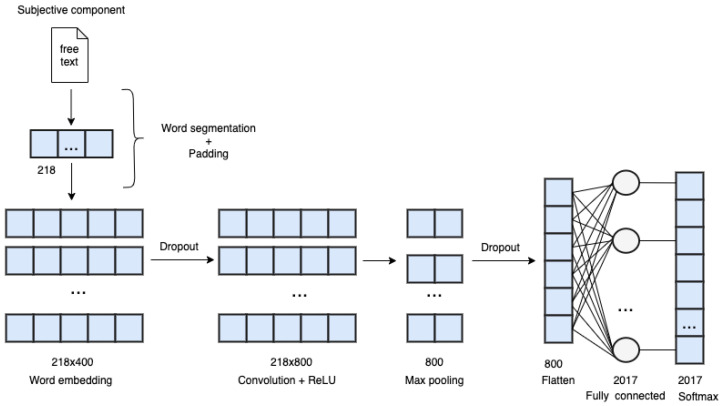
The architecture of convolution neural network.

We designed a convolutional network model by only using the subjective component of progress notes in the medical record to predict ICD-9 codes. Figure 9 shows the architecture of the Convolution Neural Network. In this project, the data cleaning and pre-processing were performed in Python, and the deep neural network was built using the Keras Python deep learning library running on top of the TensorFlow deep learning framework. The performance was evaluated by ten-fold cross-validation, with accuracy and loss recorded. Since there are 30,236 distinct words, the one-hot encoding is not preferred due to the high dimensionality curse. Therefore, we use distributed word representations as input data. The convolutional layer is preceded by a word embedding layer, which maps each word into a fixed-length vector through a lookup table. In our experiments, the dimension was set to 400 without scarifying accuracy. The parameters of layer information in our CNN model are described as follows. The filter size was set to 5 × 400, and the stride was set to 1. We choose ReLU as the activation function for excluding less obvious features. To further extract the most significant features, we added a Global Max Pooling Layer after the convolution layer. We add the Batch Normalization mechanism [27] before the output layer to normalize the input data, thus avoiding insensitivity caused by extreme values passing through the activation function. When training our model, we used Adam Optimizer [28] with a moderate learning rate to avoid overfitting. To further prevent overfitting, we added the Dropout mechanism to the convolution layer and the output layer. The rate was set to 0.5, so the model would converge moderately. A high epoch was used to give the model sufficient time to complete convergence.

### 2.4. Evaluation

**Figure 10:**
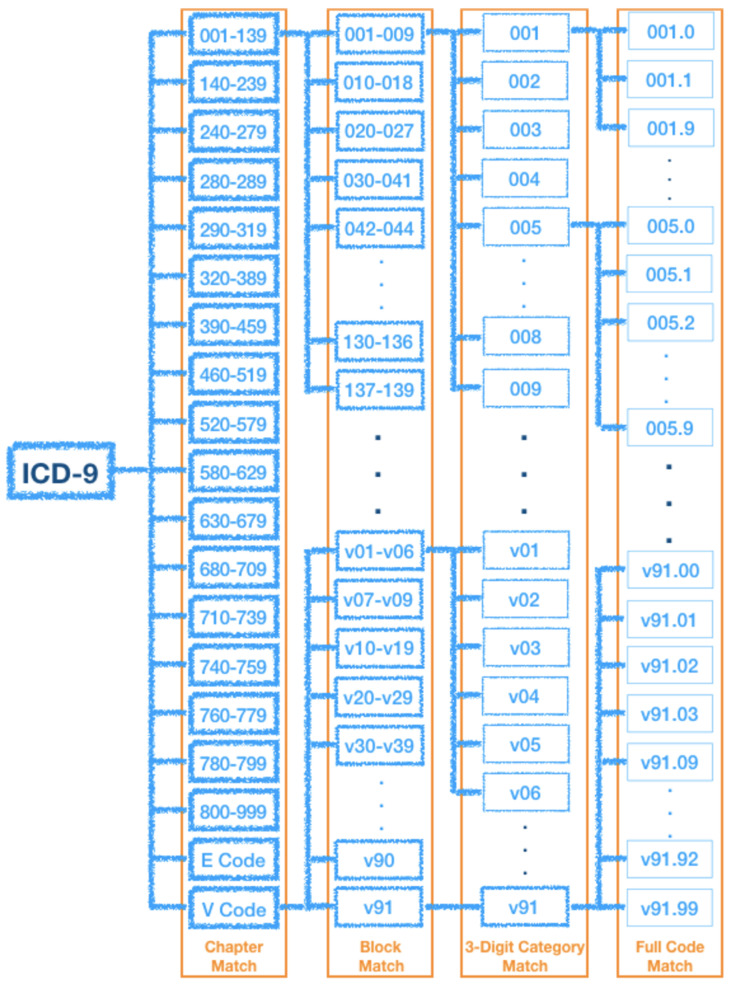
The hierarchy of ICD-9 code and match criteria.

**Figure 11:**
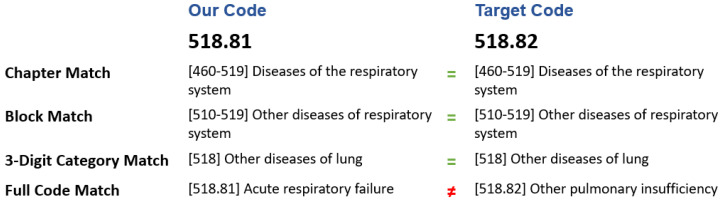
An illustration of four cases of ICD-9 match criteria.

As described in the previous section, the hierarchy structure of ICD-9 code consists of Chapter, Block, Category, and Full Code. Since there are more than ten thousand distinct codes, we may not expect that the prediction model is always able to determine the exact full code. We believe that the predicted results will be still valuable for assisted diagnosis if only parts of the ICD code match. Therefore, we reconsider the “match” criteria (i.e., the concept of correctness), and provide four cases of matching, from general to rigorous: Chapter Match, Block Match, Three-Digit Category Match, and Full Code Match, as shown in Figure 10. For example, the Chapter Match rule is as follows: if the predicted disease code and the actual code belong to the same chapter, we treat them as belonging to the same category (i.e., the predicted result is correct subject to the Chapter Match rule). Similarly, other matched cases are redefined accordingly. In Figure 11, providing that the predicted code is 518.81 and the actual code is 518.82. They are determined as a matched case in Chapter Match, Block Match, and Three-Digit Category Match, but not in Full Code Match.

## 3. Results

Table 2 summarizes the prediction performance of our CNN-based approach by using the 10-fold cross-validation. The “baseline” is calculated by dividing the number of data in the category with the largest number by the total number of the dataset.

Regarding the baseline performance, in this study, we make use of the majority class classifier as a benchmark for other learning methods in the performance study [29]. A baseline is a method that uses heuristics, simple summary statistics or randomness to create predictions for a dataset. The most primitive of all the classifiers is the naïve classifier (the ZeroR in Weka, or the dummy estimator in scikit-learn) which simply predicts the majority class in the training data set if the class is categorical and the average class value if it is numeric. Although it makes little sense to use this scheme for prediction, it can be useful for determining a baseline performance as a benchmark for other learning schemes [29,30]. In particular, the majority baseline always predicts the majority class in the data set, and the random baseline makes a random selection.

For a better illustration of prediction performance, we visualize the normalized confusion matrix, in which each cell A(i,j) is normalized by dividing the corresponding row A(i,*).

**Figure 12:**
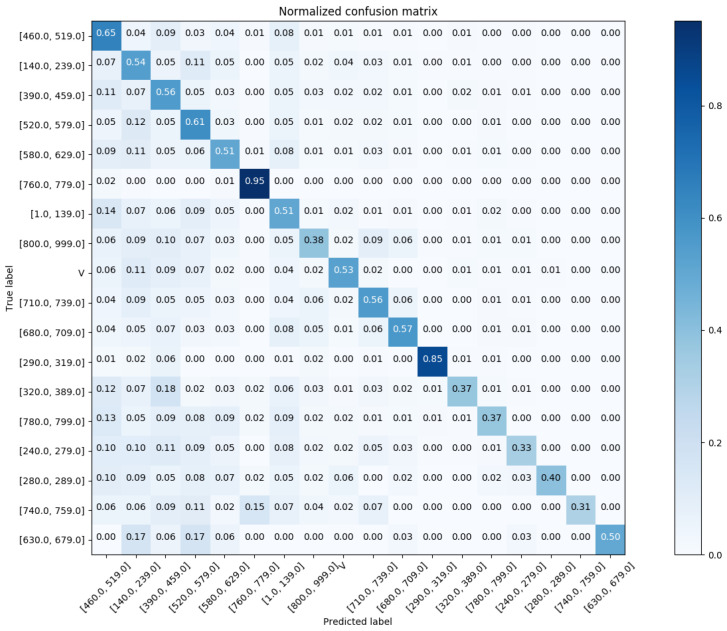
The normalized confusion matrix (Chapter Match).

**Figure 13:**
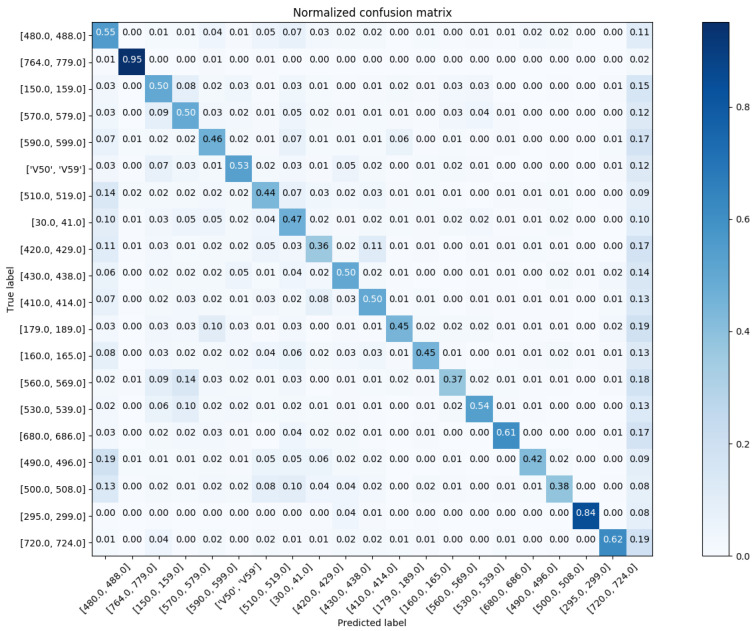
The normalized confusion matrix (Block Match; the top-20 most prevalent results).

**Figure 14:**
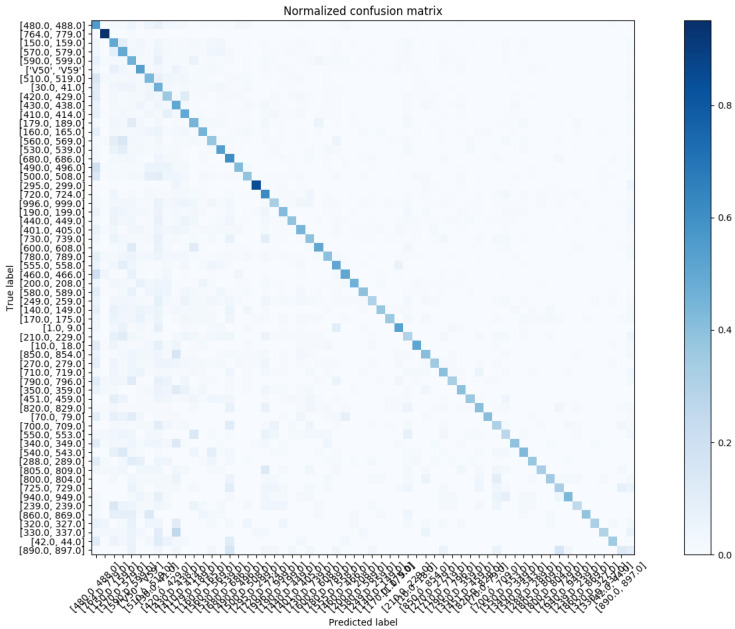
The normalized confusion matrix (Block Match; the top-60 most prevalent results).

In reference to Figure 12, we show the confusion matrix with respect to Chapter Match. Regarding the Block Match, we show the confusion matrix for the top-20 and top-60 most prevalent results in Figure 13 and Figure 14, respectively.

**Figure 15:**
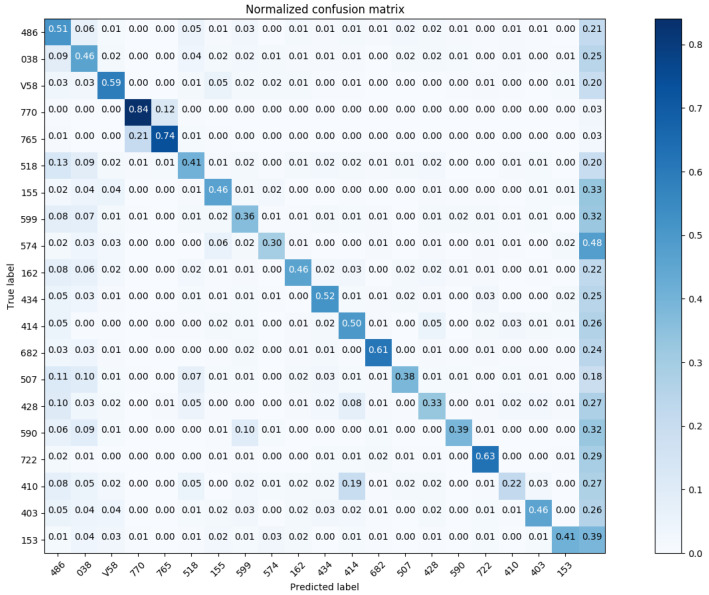
The normalized confusion matrix (Three-Digit Category Match; the top-20 most prevalent results).

**Figure 16:**
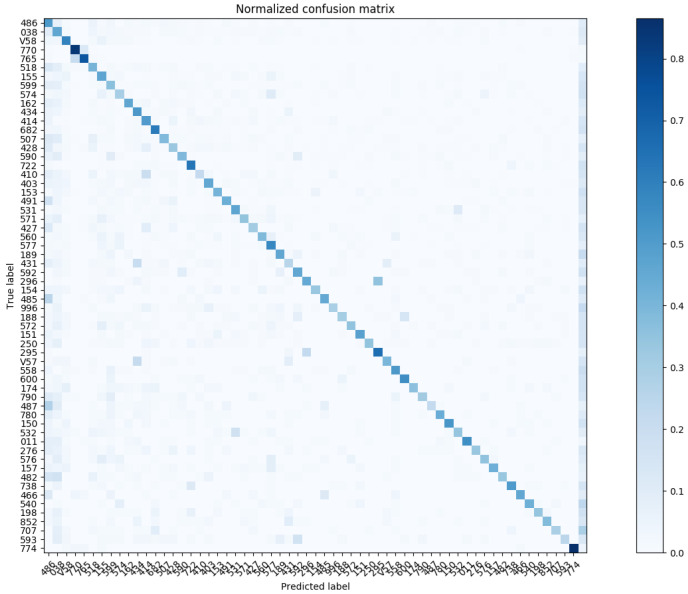
The normalized confusion matrix (Three-Digit Category Match; the top-60 most prevalent results).

**Figure 17:**
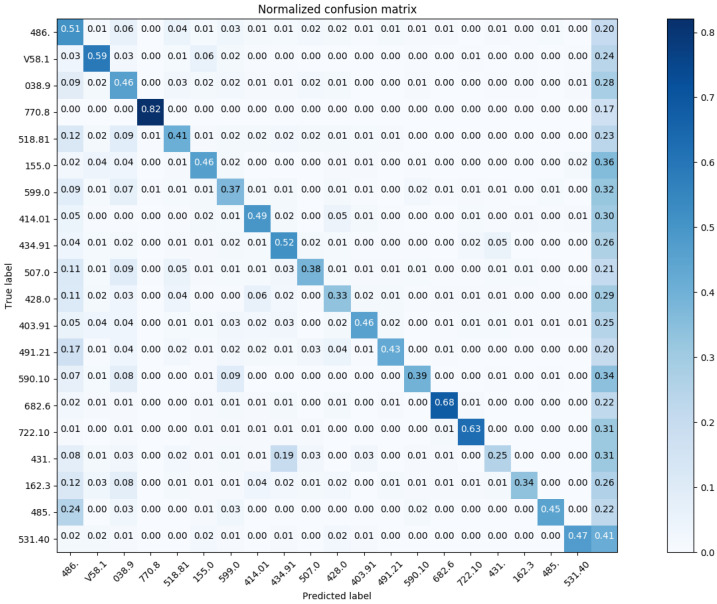
The normalized confusion matrix (Full Code Match; the top-20 most prevalent results).

**Figure 18:**
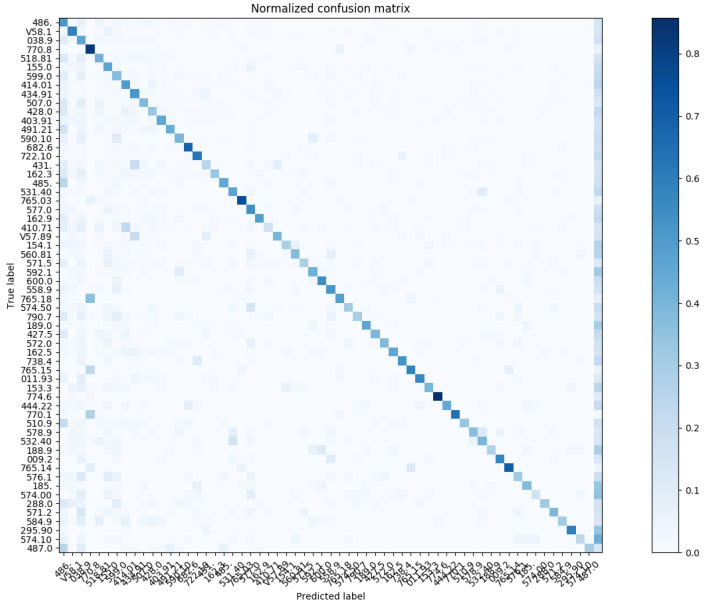
The normalized confusion matrix (Full Code Match; the top-60 most prevalent results).

Similarly, we show the confusion matrix for Three-Digit Category Match in Figure 15 and Figure 16; and the confusion matrix for Full Code Match in Figure 17 and Figure 18.

### 3.1. Case Study

**Figure 19:**
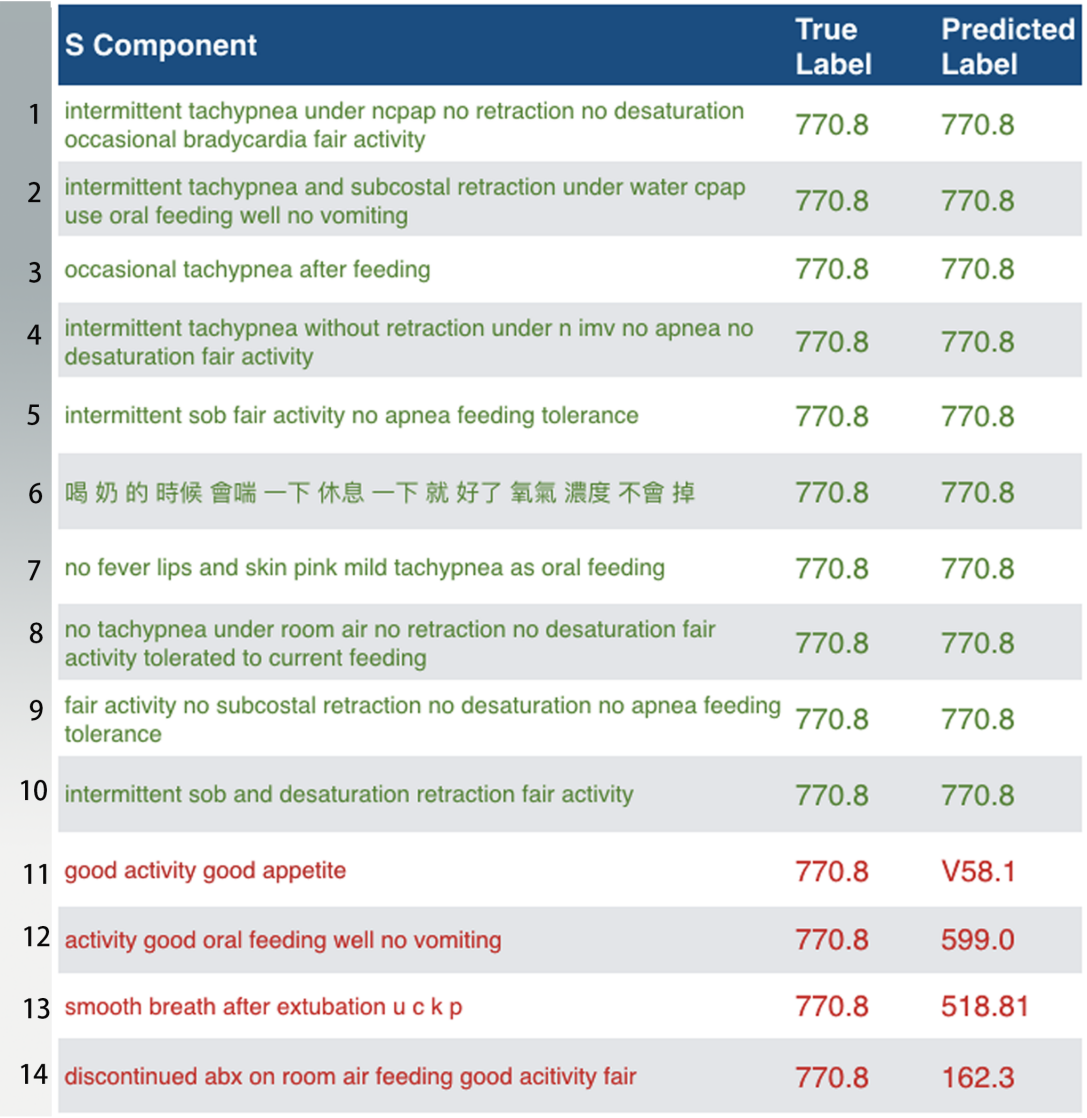
The predicted results and actual results with respect to ICD-9 code 770.8 (the rows in green are correct; the rows in red are incorrect). 6. The (original) The Subjective Component in this example was written in Chinese. The translation is “Panting during feeding. It’s fine after a short break. The oxygen levels stay the same”.

**Figure 20:**
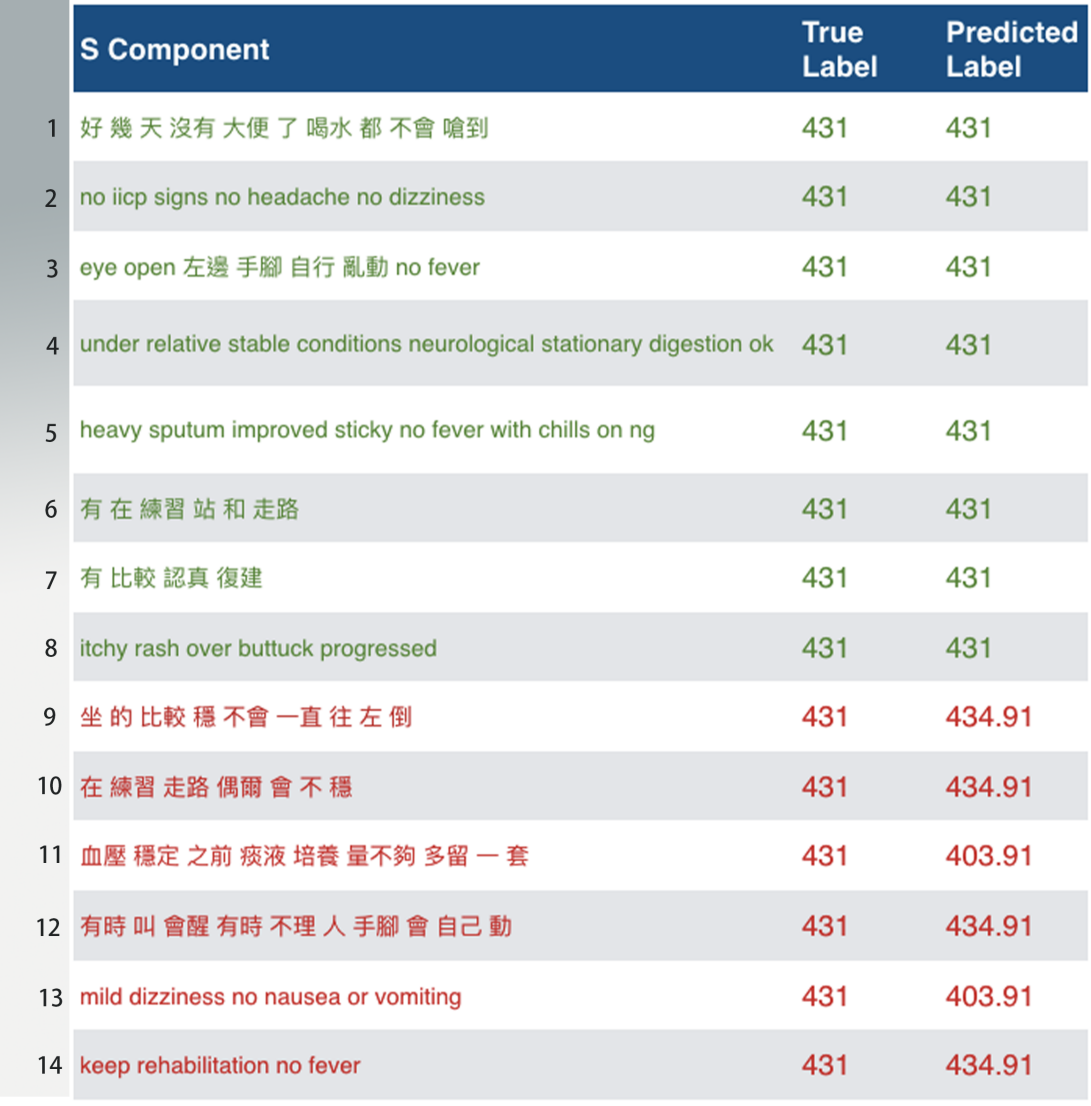
The predicted results and actual results with respect to ICD-9 code 431 (the rows in green are correct; the rows in red are incorrect). The (original) Subjective Component in this example was written in Chinese. The translation is ‘1. constipation for days; do not choke on water’, ‘3. eye open; moving left limbs; no fever’, ‘6. have been practicing standing and walking’, ‘7. have been doing rehab’, ‘9. sitting more stably without leaning to the left’, ‘10. have been practicing walking but still a bit shaky’, ‘11. blood pressure is stable; not getting enough material for sputum culture previously; saving one more set’, ‘12. sometimes wake up to someone’s calling and sometimes not; limbs are movable’.

Based on the results in Figure 17 (Full Code Match; top-20), the disease predicted with the highest correctness is ICD-9 770.8 (respiratory illnesses after birth), while the one predicted with the lowest correctness is ICD-9 431 (intracerebral hemorrhage). Among 435 medical record data of ICD-9 770.8, there were 357 correct predictions versus 78 incorrect predictions. Among 139 medical records of ICD-9 431, there were 35 correct predictions versus 104 incorrect predictions. Figure 19 and Figure 20 detail the predicted results and actual results with respective to ICD-9 code 770.8 and 431.

We further investigated the data of ICD-9 code 770.8, and some words were found to appear repeatedly in the medical records for postnatal respiratory problems, including “intermittent”, “tachypnea”, “retraction”, and “feeding”, resulting in a higher degree of similarity among the records, thus the model has a high probability of correct classification based on these high-frequency words. These highly repetitive medical records also increase the accuracy of model predictions. In addition, after reviewing the 435 medical records for postnatal respiratory problems, we found that only one of the records was recorded in Chinese, and that this record was correctly classified by the model. Thus, though Chinese language medical records only account for a very small proportion of the data for respiratory problems, the model training was still sufficient for correct prediction.

Considering the data of ICD-9 code 431, for the intracerebral hemorrhage records, we found that, in addition to the low degree of similarity of these records, these patients’ chief complaints were fairly brief and simple, using a small number of words to describe their symptoms (the records for postnatal respiratory problem prominently featured tachypnea and words related to respiratory distress).

Physicians pointed out that, because reports of intracerebral hemorrhage are typically written during patient recovery, the report content is mainly based on the condition of patients’ rehabilitation, with little direct connection to the patient’s disease, resulting in the model being unable to identify the disease.

### 3.2. Discussion

In the preprocessing for the subjective component, we filter out all non-Chinese or non-English characters, including numbers and symbols. The medical records include many numerical data such as body temperature, blood pressure, wound size, and duration of illness. The symbols used also provide important information such as positive or negative values. We hope that future work might be able to retain these numbers and symbols, indicating certain information, as input data for the algorithms. Regarding the word segmentation, there are some improvements as our future work. First, in the study, the subjective component primarily derives from the patients’ oral description of their conditions and contains some medical terminology (e.g., medical examination items, field names). However, at present, our dictionary files are not optimized to perform word segmentation for medical proper nouns which may be incorrectly segmented or mixed with other irrelevant words. The accuracy of our approach could be further improved by enriching the dictionary file of word segmentation with medical terminologies. Second, we are currently using a Chinese dictionary file as the primary basis for word segmentation. However, some patient self-report descriptions had been directly translated into English by medical staff or physicians, when composing the subjective components. The Jieba word segmentation tool used for this study breaks English words only on the basis of blank spaces, resulting in poorer performance on complex English words (including abbreviations). We hope that future work could use specialized English-language word segmentation tools to improve the segmentation performance.

Currently, we make use of standard text processing, including lower-case rendering and word segmentation. In such case, the digit will be segmented. For example, as we have a segmented word “39”, we cannot be sure about the semantics of word: whether the “39” is body weight or body temperature without context-sensitive information (e.g., the previous and/or the adjacent word of “39”). That is the reason why we did not take digits into consideration in this pilot study. In the future work, we will use *n*-gram (e.g., bigram or trigram) rather than a single word (i.e, unigram) used in this study.

In the original medical record, the medical staff or the physician will perform a simple test on the patient and record the result after the patient’s complaint data. For example, if the patient has been injured in a car accident, medical staffs will usually record whether or not the patient had been wearing a seat belt or a crash helmet, with results indicated by “是”, “否”, “YES”, “NO”, “+”, “-”, etc. However, the word segmentation tool will fail to connect the test item with the result, resulting in a loss of test data that may be relevant to the patient’s condition. That is, we need a semantic word segmentation and text preprocessing to resolve context-dependent descriptions of self-report texts.

Once, we used the Google Cloud Translation API, a translation tool, to translate the English text in the medical records into traditional Chinese text, and then performed word segmentation with Jieba. We had hoped to integrate some Chinese and English synonyms through the translation process and thus reduced the number of dimensions in the medical records, but later we found that this did not increase the predictive accuracy. In addition, we eliminated stop words, both the commonly used stop words and those used in medical contexts, but this also did not improve prediction accuracy, possibly because the original high-dimensional data contains more implicit information. In the end, we removed these two programs to retain more complete information. When adjusting the height of the convolution layer filter to find the filter size with the highest accuracy, gradually increasing the size, we found that optimal accuracy was obtained with a convolutional layer filter size of 5 × 400. That is, the best convolution is of five words. In addition, we found that the maximum number of words for the input data is also five. Previously, designing neural networks often required adjusting parameters one by one to find the best model. Perhaps this process could be made more efficient by observing the maximum number of words as a reference for adjusting the filter height, so that the time of adjusting the test one by one can be saved.

## 4. Conclusions

In this paper, we build a CNN-based model to predict the ICD-9 code by only using the subjective component of medical records. The average accuracy obtained by 10-fold cross-validation is 0.409. If considering the “chapter match”, the prediction performance of our model is with a recall of 0.58, precision 0.582, and accuracy 0.580. Currently, the performance of prediction is promising, but has not yet reached a standard at which it could be put into practical use. The future work will focus on improvements mentioned in the previous section and the integrated approach by using both subjective and objective components of medical data.

## Figures and Tables

**Table 1:** The summary of ICD prediction works.

Work	Methods	Data	Performance
Gangavarapu et al. [16]	Fuzzy similarity-based data cleansing approach, supervised multi-label classification models	Nursing notes	Accuracy of 0.82 (with respect to 19 distinct ICD-9 chapter code)
Gangavarapu et al. [17]	MLP, ConvNet, LSTM, Bi-LSTM, Conv-LSTM, Seg-GRU	Nursing notes	Accuracy of 0.833 ( w.r.t. 19 distinct ICD-9 chapter code)
Samonte et al. [20]	Enhanced Hierarchical Attention Network	Discharge summary	Accuracy of 0.841; Precision of 0.780; Recall of 0.620; $F_{1}$ score of 0.678 (w.r.t. 10 distinct ICD-9 code)
Moons et al. [21]	CNN, GRU, DR-CAML, MVC-LDA, MVC-RLDA	Discharge summary	Micro $F_{1}$ of 63.42%; Macro $F_{1}$ of 59.74%; Micro AUC of 91.57% (w.r.t. the most frequent 50 distinct ICD-9 block code)
Hsu et al. [22]	CNN, LSTM, GRU, Attention	Discharge summary	Micro $F_{1}$ of 0.76 (w.r.t. 19 distinct ICD-9 chapter code); Micro $F_{1}$ of 0.57 (w.r.t. top-50 ICD-9 code); Micro $F_{1}$ of 0.51 (w.r.t. top-100 ICD-9 code);
Obeid et al. [25]	DNN, CNN	Clinical notes: progress notes, plan of care notes, emergency department provider notes, history and physical notes, and consult notes	AUC of 0.882; $F_{1}$ score of 0.769 (w.r.t. ICD-9 code from E950–E959)
Huang et al. [19]	Logistic regression, Random forests, Feedforward, NN, CNNs, LSTM-RNNs, GRU-RNNs	Clinical notes: medical history of patients, patient’s comments during the interaction, doctor’s observation note	Average precision of 0.3757 (w.r.t. top-50 ICD-9 codes); Average precision of 0.4565 (w.r.t. top-50 ICD-9 category code)
Chen et al. [18]	CNN-MDRP	Demographics of the patient, daily habits, examination elements, results, diseases, patient’s readme illness, and doctor’s records.	Accuracy of 0.948, w.r.t. chronic disease prediction
Suo et al. [23]	Time-fusion CNN	Diabetes patient cohort, obesity patient cohort, and chronic obstructive pulmonary disease (COPD) patient cohort	Diabetes accuracy of 0.8009; Obesity accuracy of 0.8737; COPD accuracy of 0.8133
Cheng et al. [24]	CNN based model	The congestive heart failure (CHF) patient cohort and COPD patient cohort	CHF accuracy of 0.7675; COPD accuracy of 0.7388.
Our work	CNN based model	Subjective component	Accuracy of 0.580 (w.r.t 17 distinct ICD-9 chapter code); Accuracy of 0.409 (w.r.t. 2017 disticnt ICD-9 code)

**Table 2:** Prediction performance of our approach.

Rule (Match Criteria)	Recall	Precision	F1-Score	Accuracy	Baseline
Chapter Match	0.580	0.582	0.579	0.580	0.162
Block Match	0.494	0.503	0.492	0.494	0.081
Three-Digit Category Match	0.433	0.453	0.431	0.433	0.061
Full Code Match	0.409	0.436	0.404	0.409	0.061
